# The social dimension of biobanking: objectives and challenges

**DOI:** 10.1186/s40504-017-0059-5

**Published:** 2017-09-13

**Authors:** E. V. Bryzgalina, K. Y. Alasania, T. A. Varkhotov, S. M. Gavrilenko, E. M. Shkomova

**Affiliations:** 1 The Department of Philosophy of Education, Faculty of Philosophy, Lomonosov Moscow State University, Moscow, Russia; 2 The Department of Philosophy of Politics and Law, Faculty of Philosophy, Lomonosov Moscow State University, Moscow, Russia; 3 The Department of Philosophy and Methodology of Science, Faculty of Philosophy, Lomonosov Moscow State University, Moscow, Russia; 4 The Department of Ontology and Epistemology, Faculty of Philosophy, Lomonosov Moscow State University, Moscow, Russia; 5 The Department of Ethics, Faculty of Philosophy, Lomonosov Moscow State University, Moscow, Russia

**Keywords:** Society social and humanitarian support biobank biobanking social technologies bioethics biotechnologies

## Abstract

The present article allows to explore, analyze and reflect on the consequences and problems posed by biobanks and attempts to prove the need of social and humanitarian support in establishing and functioning of biobanks as a new type of scientific institutions. The basis of the article is the latest publications devoted to social and humanitarian aspects of biobanking and Russian experience of the initial formation of this subject domain (before the first professional biobanks were established in Russia in the 2010-s, the only highly specialized collections of bio-samples had been registered). The article marks and classifies different aspects of biobanking that objectively demands the participation of specialists in ethics and social sciences. The cases of biobanking development and risks are estimated; the objective need of applied ethics and social sciences specialists’ participation in biobanking is proved.

## Introduction

The development of individual and translational medicine as well as a still unsolved issue of conceptual interpretation of the human genome make modern research in biological chemistry, molecular, cell biology, and other allied scopes impossible without collecting, storing and using different types of biological material. Establishing banks-depositories of biomaterials of any type, and especially of the human material kind is connected with the need of solving a range of complicated social and humanitarian issues.

Biobank activities, ultimately, are the transformation of individual bodies into biological data, in other words, conversion of the unique, individual and – in the case of human being – personal into typical (serial), representative, species. In the case of banks storing human biological samples and data, this function objectively needed for a modern complex of biological sciences and technologies is associated with serious difficulties of the ethic and social, in general. The first ethic problem arisen was the fact that a new kind of natural resources – human biological material – is inside the human being body, in other words, in creatures being personalities that have certain inalienable rights. Secondly, alliance of biotechnology with informational and communicational technologies discovered the human body represented as the data, which provoked the concerns for the data protection, confidentiality and privacy. Thirdly, taking into consideration potential economic, technological and scientific value of the human being biological material and bio-information correlated to it, politicians were concerned about “genes’ theft” or unauthorized bio-surveillance over incompetent individuals (Karlsen & Strand 2009, 576).

Hereinafter, we analyze consequently the key directions of the biobank development that demand social and humanitarian support due to various reasons. Not all of them are recognized as demanding the participation of experts in social sciences. And many of them are considered as the domain of independent professional competence of sellers and consumers of the biobank services. Further, we address to Russian experience (that is limited yet because of the initial stage of biobanking in Russia), show the challenges the biobank development faces in Russia and the need of social and humanitarian support in this sphere today.

Biobank development analysis shows that we deal with the object which is not completely formed. This object is heterogeneous and highly complicated, it includes technical, economic, communicational, law, ethic, social and political components and each of them has its own issues demanding an integrated mutually agreeable solution in terms of each specific bank. Herewith, a range of questions inevitably arising in the process of the biobank activity depending on decisions that go beyond technical competence of subject specialist (technologists, medical professionals, biologists, lawyers etc.) and demanding a wide-range inter-sector discussion with the assistance of social sciences specialists.

## The issues of social and humanitarian support of biobanking

Two main groups of issues demanding social and humanitarian support can be marked out:

1. the issues of ethical and law regulations of the biobank activities allied to the forms of necessary participation in social and political life and the risks of this kind of participation. These issues can be called “outer” ones, because they concern the forms of biobank activities where it is not completely autonomous (relations with donors, estimation of social acceptability of research objectives and methods when biobank materials are used, the use of research results in decision-making that concerns the health system organization etc.).
2. the issues of organizing communication and marketing activity of biobanks. These questions can be called “inner” ones as far as they concern functional support of the biobank considered an economic entity.

At first sight, “inner issues” are of technical character and refer only to the competence of owners and organization management. In fact, it is not the case, because a range of key components of technological and administrative support influence considerably the perspectives of its use and causes specific social (economic and political) risks going far beyond “inner” issues of administration and technological support. This kind of components may include:

1. The choice of storage technology and types of biological materials designed for arrangement in a depositary, by reference to usage perspectives. A specific decision made on these questions while establishing the biobank project considerably restricts not just objectives of the biobank collection further use, but options of collaboration with other biobanks and participation in the research using materials and data of different biobanks. Meanwhile, the demand for such kind of research increases because of rational and technically implemented transit to the methods of individual medicine and, accordingly, dependence of corresponding scientific methods on a sampling size and increasing need for new samples (Elger & Biller-Andorno, 2010). Significantly, the members of European biobanking industry frequently consider the willingness to the data exchange and collaboration with operators of other collections to be an important part of defining the biobank, which distinguishes this object from just a sample collection (Master et al. 2015, Shaw 2014). Moreover, the choice of a technological model in biobank organizing determines an economic model of its functioning in many respects, including current financing (infrastructure support cost) and commercial perspectives. That is why relevant decisions should be made by operators and administrators together with specialists of economic forecasting, science ethics and science sociology.
2. The organization and support of the data base of sample information management.
  The efficiency of biobank functioning regarded as a source of material (samples and data) for scientific and clinic research considerably depends on completeness and quality of information massive connected with samples – its relevancy, completeness of phenotype and genotype description (for clinic research), parametric efficiency and sociometric data validity (for population research). However, forming and supporting the data massive demands solving both traditional methodological problems of collecting sociological data (efficiency of the basic parametric matrix, intentional and unintentional data mispresentation made by donors, information hiding etc.), and specific communication problems arising when working with donors (the ethics of asked questions, forming donor’s willingness to provide confidential information, providing donor’s availability for up-dating etc.) The listed problems determine two tasks obviously demanding social and humanitarian support: methodological support of the biobank activity in terms of ethic, law, social and psychological instruments as well as forming individual and collective confidence in biobanks as information consumers and distributers. The design of a model of informational and sociological management of material collection demands the participation of a wide range of information specialists: from subject experts being the biobank data base consumers to ethics specialists, professionals in the spheres of information collection methods, psychological work and public relations.
3. Developing a model of communication availability: the principles of regulating an access of outside subjects to the biobank data base.
  As far as biobanks are designed for research objectives, a certain level of information availability is a natural premise of their functioning. However, because of information peculiarities contained in the biobank data base, there is a risk of non-ethic and even criminal use of the data by the third-party agents. For instance, for insurance companies specialized in medicine, the biobank data bases are an essential tool for risk estimating and assurance cost forming. For pharmaceutical companies, it is both a set customer base and an excellent marketing tool. However, biobank donors hardly agree to medical insurance cost and medications’ increase being a side effect of their own consent to samples’ providing. It should be considered that the flip side of information availability is an opportunity of the data abuse. It means, the issues of regulating data and communication policy of biobanks should be thoroughly verified. Biobanks are functioning, when society “genetising”. This process is characterized by increasing attention to genetic discoveries when population lacks knowledge for understanding relevant information meaning. While lacking specialists for personal consulting, the danger of stigmatizing certain social groups and/or individuals increases, proceeding from the data associated with them.

There are even more obvious biobank development problems demanding social and humanitarian support. Among them, there are multiple ethic complexities and other issues of “outer” activity management allied to them.

1. One of the oldest problems of this kind is a form of donor consent to use provided samples (Hofmann et al. 2009, Serepkaite et al. 2014). A classic model of regulating the use of human materials for research objectives is the “informed consent” received by a researcher from a patient, confirming commitment to provide personal biological material for a certain kind of the research as well as a decision of a bioethical commission (an ethic committee) made about moral acceptance of the conducted research objectives and methods. In the case of biobanks, the use of classical understanding of the informed consent is quite challenging because of objective ambiguity of further use of the received donor material and corresponded information (biobanks imply multi-target long-run use of samples). Another reason is interpretation of the term “informed” in terms of receiving samples for a non-specific experiment (Master et al. 2015). A classic procedure of the informed consent implies informing the donors about the fact that they are free to refuse the consent given by them and terminate their relations with the biobank at any time with no personal charges. However, this kind of relations is not objectively comfortable for prominent players of biobanking industry.
2. Updating the donor consent before each manipulation with the material seem to be complicated because of logistic and financial reasons as well as it is inconvenient for the donor that has to go repeatedly into details before document signing and frequently has no opportunity to estimate thoroughly the conditions listed in the document to be signed. In this respect, the experience of the foreign countries shows that this problem is in the process of solving. The use of different variants of the basic (universal) consent including the basic consent having the limits of spheres of sample use (Master et al. 2015) is suggested. Besides, non-standard solutions in the mood of solidarity ethics, the social good and collective consent are suggested (Karadi 2011). Measures appropriately protecting the rights and dignities of donors being unable to give their legal consent demand a special discussion (children (Giesbertz et al. 2014), mentally disabled persons (Togni et al. 2014) etc.).

In spite of diversity and significant differences in radicalism of suggested solutions, all of them have a trend to legalize disposal of biological material from donors. In the certain final perspective, it means ignoring the fact that “I” is identified with one’s personal body and transmitting the body to other persons, which means objectification of the corporal human being in a form of “biological material” having a development perspective of appropriate modifications (total) of the social order.^1^ In fact, the procedures of anonymizing (donor’s individual data assuring personal life privacy and inviolability) had a double effect: in practice, they were used to limit the need of the repeated consent; symbolically or rhetorically, they were a strategy for supporting the climate of the public consent. The de-identification of subject (who is getting anonymous and its disposal from biological samples and data is taking place) was unobvious allied with losing the individual interest towards biological materials and data they contain. As a result, the idea of the donor refusing any form of material control developed. That let market agents adopt biological materials as res nullius. Consequently, the identification of subject and the power of controlling biological materials were linked clearly (Tallacchini 2015, 24–25).

3) In terms of biobank functioning, there is an objective conflict of interests in linked social groups (donors, biobank holders, scientists the users, state power and commercial organizations). Each audience is objectively interested in expanding the biobank control. As far as the interests of audience in the sphere of demands presented to biobanks and principles of their functioning do not coincide, it comes to contradictions that cannot be eliminated completely. And that is why they must be thoroughly regulated. For instance, donors are interested in providing their privacy and informing in order to control the use of the biobank materials (inserting ethic and other regulations of an access to samples in connection with personal moral and religious views, social and personal status change, family attitude influence etc.) as well as in participating in commercial use of biobank funds. The biobank holders are interested in disposal of materials from donors in order to expand the sphere of free use of funds, develop project activities, reduce expenses for getting the consent, monitoring the correspondence between the received consent and new variants of data use, for work with donors. Scientists are interested in materials’ availability, standardization of information and technological parameters of biobank organization, simplification of a procedure of receiving donor consent. It is obvious that fulfilling all the listed requirements is impossible. The interests of parties are controversial, so, for the biobank successful functioning, the development of a regulatory model taking into consideration and, at the same time, limiting the interests of all the audience is needed. This model should also work in terms of perspective risks connected to implementing or non-implementing these interests.
4) Elaborating models of biobank commercialization and profit distribution is a specific issue.^2^ For example, to get medication in the case of using materials of a rare disease carrier (frequency: not more than 1 per 500 persons) or orphan disease (frequency: not more than 1 per 10.000 of population) the moral right for donor’s participation in profit-sharing is described. In the case of obvious commercial perspectives, should the samples be purchased by the fixed price, or can donors claim for the rent during the period of participation in biobank functioning, or is it necessary to provide donors with participation interest, sharing the risks of materials’ commercialization and the results of their use? The choice of an economic model, from the point of fair interest balance and estimations of participants’ contribution into biomedical technology development, and the choice of an effective model of biobank commercialization as well as the issues listed before cannot be properly implemented without involving the experts specialized in ethics and social sciences. And they should be a part of a grand complex of measures of social and humanitarian support of the biobank project development and functioning.

## Biobanking development: the case of Russia

Unlike western countries, where the process of biobanks’ establishment began at least in the 1990-s, biobanking in Russia is a new phenomenon that is at the stage of formation. At the moment, a few big biobanks function in Russia – the bank of biomaterials of the Federal State Budgetary Institution “East-Siberian Scientific Center of Human Ecology” (Siberian Branch of the Russian Academy of Medical Sciences), in 2012 the biobank of V.A. Almazov North-Western Federal Medical Research Center started functioning. At the EuroQSAR symposium in March 2014, the successful start of the first commercial biobank in Russia called “NBS” (National Bio Service) was announced.

The Russian Federation is the participant of the international project on establishing the biobank studying thyroid cancer. The event encouraged this project was the tragedy of Chernobyl Nuclear Plant in 1986. The exhaust and formation of radionuclide of iodine caused mass radiation exploration of the thyroid in Ukraine, Russia, Belorussia. In 1998, the European Commission, World Health Organization, National Cancer Institute and Sasakawa Memorial Health Foundation came forward with an initiative of the international project of establishing the Chernobyl bank of thyroid tissues, blood, nucleic acids and data base. The activity field of this bank would be collecting unique material and information that is significantly important for health gain of future generations. The foundation of the bank was supported by Belorussia, Ukraine and Russia. The locations of the bank are Minsk, Kiev and Obninsk.

At the present time, the foundation of a biobank that would focus on collecting materials of “all the living organisms on Earth” is a scientific project of Lomonosov Moscow State University. This project is symbolically titled “Noah’s Ark” (National Depositary Bank of Living Systems). This project should lay foundation for forming unique policy of biobanking development based on interdisciplinary specificity having a wide range of research goals. So, Moscow State University serves as a scientific center having the key objective of elaborating scientific basics of biobanking.

However, there are other aspects of biobanking development besides scientific purposes. This fact is determined by heterogeneous concept of the biobank. And the aspect of law regulations of biobanking is of crucial importance.

When speaking about peculiarities of biobanks’ regulations in Russia, we note that biobanks’ activities are regulated by separate laws and subordinate acts. There is no special law created in accordance with the new Constitution.

The law basis forming in Russia is built on international law acts such as the Universal Declaration of Human Genome and Human Rights (UNESCO, 1997), the International Declaration on Human Genetic Data (UNESCO, 2003), Convention for the Protection of Human Rights and Dignity of the Human Being with regard to the Application of Biology and Medicine; World Medical Association Declaration of Helsinki on Ethical Principles for Medical Research Involving Human Subjects; International Guiding Principles for Biomedical Research Involving Human; the Universal Declaration on Bioethics and Human Rights etc. But the norms of these acts serve only as guidelines and do not determine the specific aspects of everyday biobanking’s practice.

Among main federal laws that can be potentially recognized as the platform when implementing the project of Moscow State University “Noah’s Ark” are the Federal Law of 27/07/2006 # 152 “On personal data”. This law is the important guarantee of the constitutional right of the individual for non-interference in privacy from third parties; the Federal Law of 03/12/2008 # 242 “On the Government Genome Registration in the Russian Federation”; the Federal Law of 05/07/1996 # 86 “On the Government Control in Gene Engineering Activity”. Among subordinate acts, the following should be mentioned: the Russian Federation Government Edict of 28/12/2012 “On Adopting the Strategy of Medical Science in the Russian Federation for the Period up to 2025”, the Order of the Ministry of Health of the Russian Federation of 30/04/2013 #281 “On Adopting the Scientific Foundations of Medical Science”, the Order of the Federal Agency on Technical Regulating and Metrology of 14/04/2014 #472 “On Establishing a Technical Committee of Standardization of Biotechnology” and the Draft Regulation of the Russian Federation Government “On the Program of Fundamental Scientific Research in the Russian Federation in a Long-Run Period”.

It is to be noted that in the nearest time, a law gap will be partly filled, because, on the 23/06/2016, the President of the Russian Federation endorsed the law #180-FL “On the Biological Cell Products”. This law will take effect on the 1st of January, 2017. This law regulates the relations including those of “storage and transport of biological materials”.

Consequently, law regulations of biobanks are fragmentary. At the moment, unlike a range of western countries, there is no unique law regulating biobanks’ activities from the stage of establishment to the stage of sampling, storage and possible transfer of biomaterials.

The example of Russia, where organization, law and media fields of professional biobanks’ activity are forming intensively, clearly demonstrates heterogeneous character of biobanking that function “at the edge of genomics, ethics, social and law profit”.^3^ When having no set system of normative and law regulations and interpretation of biobanking at the level of public opinion, practically all key aspects of biobank activities – form of informed consent, principles of interactions with donors, policy of providing users with materials’ collections, selective policy in the case of national bio-depositories of the “Noah’s Ark” kind, information policy etc. demonstrate the presence of social implications closely connected to the questions of biopolitics and “social interests”.

For instance, one of the most promising directions of biobanking application is the development of personalized medicine (Eropkin 2015, 11). Personalized medicine is considered to be a step in the direction of high value medicine and one of the ways to economy of knowledge. However, along with impressive advantages of individual adjustment of diagnostics and pharmacology, there are rather impressive risks of regeneration of eugenics and different forms of discrimination on biomarkers’ grounds – beginning from changes in cost of medical insurances to forming new tools of the state biopolitics and population government. These risks are integral parts of biobanking institute development and they demand participation of specialists in social sciences.

When having pragmatic advantages connected with local engineering (research) purposes, final outcomes of biobank activities, including those of social consequences, are determined by implicit target-setting and ethics principles. The choice of these principles cannot be made in automatic mode. This choice cannot be also trusted in full to engineers, businessmen and natural scientists. The experts in social and humanitarian support should participate at all stages inasmuch as we deal with a new fundamental social institute formation.

## Conclusion

Exploring brand new space and territories, new types of products and services, production and economic exchange, practices of consuming and life styles can model the new market forms and, potentially, new anthropology corresponding to them.

In connection to biobank industry development, it is possible to speak about developing new strategy forms intended to “invade” into the individuality of human being body at the deepest (polymorphous and molecular unique) level, using available individual biomaterial samples as new bearing points and new biotechnologies as new tools for providing their own performative efficiency.^4^

Although, at the moment, the application field and approbate opportunities of biobanks are not used at the global level, they are not totally controlled and they are also far from changing biological nature of the human being, a relevant opportunity is implied by the structure of the research, engineering and technical practices that were produced and used by biobanks. The development of biotechnologies and genomics can be examined as a part of social and technical regime, whose center is the new biological knowledge of the human being body (Hedgecoe & Martin 2008, 819). It should be taken into consideration that this regime can cross the borders of the industry generated it and be the basis of not only biomedical but also social and political technologies.

The analysis of biobanks’ development objectives should include the discussion about the consequences of the possibility connected with the fact that, at the basis of biobanks, the projects of eugenic kind (technical, “scientific” constructing of the “healthy and happy” body that has no physical (and, probably, other) sufferings) and the ideas of naturalization of social inequality and hierarchy regimes can reappear. At a time when technological breakthrough, local efficiency of tools and decisions are taking place, such kind of projects and ideas can possibly seem “natural” and “progressive” for at least a part of social groups.

So called “social and humanitarian support” of biobanks became a separate intellectual research abroad long time ago (primarily, in the EU and North America). From the research legitimacy and possible practical contribution point of view, it depends on understanding the complexity and heterogenic character of the biobank, comprehending the fact that biobanks are not just places to keep, store and produce the knowledge. The biobank functioning activates and assembles (borrowing the term from Deleuze and Latour^5^) different “elements” (“scientific”, “technological”, “economic”, “ethic”, “law”, “social”, “anthropological”, “ideological” etc.). The element assembles are extremely variable and changeable to much extent.^6^

The biobank is an object being at the stage of formation. Possible trajectories of its development are able to be a stake in multiple and diverse social conflicts about biobanks taking place between social agents and their specific interests (scientists, doctors, states, investors, donors, groups of civil activists).

In this context, social and humanitarian support of the biobank is crucially needed. The body and personality, biological and social in the human being, individual and social do not belong to different reality. Something that happens to one component has an essential impact on another. When investing in biotechnologies’ development, it should be remembered that biotechnologies are always social technologies and they have social consequences, because their source and object is not an abstract “biological body” but the human being having the whole variety of features, forms and definitions.

## Footnotes

1 At the beginning of modern clinical medicine, clinic patients are considered the “material” for searching means of treatment for the “society” (humanity). In this sense, personal recovery and, in general, personal destiny of certain patient was interesting just in terms of its contribution to the fundamental clinical presentation promoting the investigation of a disease and, in perspective, victory over it for the “public good”. As the director of one of the first European maternity hospitals said – the obstetrical hospital of Gottingen University (Germany, founded in 1751): “The Pregnant Women are here for the Sake of the Teaching Institution” (cit. ex: (Shlumbohm 2008, 78)).

2 A special problem arisen by biobanking in a new way is intellectual property rights. Ref.: (Hawkins 2015).

3 The quotation educes one from four fundamental aspects of defining biobanking suggested by professor Akim Abayomi from Stellenbosch University (Capetown, RSA) in Geneva at conference for biobanking in connection with epidemic Ebola on 13 of May, 2015.

4 The notions of biopolitics and biopower commonly used as research tools in the works devoted to biobanks and biobanking (for instance, (Ducournau, Cambon-Thomsen 2009) and (Karlsen et al. 2011)) originated from Michel Foucault’s intellectual heritage. The biopower is the total of mechanisms by which something that determining biological features of the human kind can penetrate into politics, political strategies, into the general strategy of power (Foucault 2009). Anyway, the subjects of donors’ informed consent (at the moment, an irremovable procedure in biobank functioning and the subject of continuing debates) and confidence in biobanks as new biotechnological institutions (Starkbaum 2014) (confidence is frequently declared a necessary condition in biobank activities) are naturally fit into Foucault’s discourse of liberal ruling regimes based not so much on radical physical violence and/or economic expropriation, but delicate mechanisms of producing consent and recognition (Karlsen et al. 2011, Mascalzoni 2015)).

5 The term “assemblage” is incorporated into intellectual discourse by J. Deleuze and F. Guattari (Deleuze & Guattari 2004). Because of their context complexity, we take the explanation from H. Watson-Verran and D*.* Turnbull: the assemblage is similar to the episteme complemented by technologies. However, the “assemblage” connotes situational ad hoc contingency of collage in terms of its capability to cover a wide range of inconsistent components. Moreover, the advantage of the term “assemblage” is connotations of active practices but not passive static strictures (Watson-Verran & Turnbull 1995). Ref. also important explanations in John Law (Law 2004).

6 The inevitable methodological complexities are caused by that. How to construct the biobank as a research object, taking into consideration its complicated nature? Basically, there is no answer to this question. The diversity of research methods and subjects of their application in social and humanitarian support of biobanking is great. The system analysis (bearing the postulates of the object entirety and its hierarchy structure organization) is not the only methodological opportunity that modern social research represents. For example, conceptual and descriptive resources of so-called «Science Technology Studies» (STS) and actor-network approaches elaborated for description, primarily, scientific and technical innovations demand detailed examination. One of theoretical functions of the “net” notion is demonstration of certain irregularity of segregating the system from the environment implied by the system method when analyzing complicated processes and objects of modern techno-science. (Ref. to STS and actor-network theory (Hedgecoe & Martin 2008), (Latour 1987) and (Latour 2005). It is significant that the representative actor-network – Latour’s “Pasteurization of France” (Latour 1988) – is devoted to the research of the biotechnological innovation – Pasteur’s invention of anthrax bacterin).
